# P14 The antibiotic resistance profile of bacteria causing acute respiratory tract infections in Khartoum, Sudan

**DOI:** 10.1093/jacamr/dlac053.014

**Published:** 2022-05-31

**Authors:** Shima Faisal, Mohmed A Suliman, Elamin Elnima, Rehab Ahmed

**Affiliations:** 1 Department of Pharmaceutics, Faculty of Pharmacy, University of Khartoum, Khartoum, Sudan; 2 Department of Microbiology, Faculty of Medical Laboratory Sciences, Sudan University of Science and Technology, Khartoum, Sudan; 3 Department of Natural products and Alternative Medicine, Faculty of Pharmacy, University of Tabuk, Tabuk, Saudi Arabia

## Abstract

**Background:**

Acute respiratory tract infections (ARTIs) are the most common indication for antibiotic prescriptions. This is associated with irrational prescription and correlated with antimicrobial resistance especially in low-income countries including Sudan. This is a cross-sectional study aimed at identifying bacterial pathogens causing ARTIs and their susceptibility to the commonly prescribed antibiotics.

**Methods:**

Bacterial pathogens (51 isolates) were obtained from 29 ARTI patients from two hospitals in Khartoum in the time period March 2021 to March 2022. Samples were collected from each patient using both nasopharyngeal and oropharyngeal swabs. They were then cultured using blood and chocolate agar. The isolated colonies were identified using Gram staining technique, biochemical test, and selective media. Antibiotic susceptibility tests (AST) were done using the Kirby–Bauer disc diffusion susceptibility test protocol. Susceptible strains were determined following CLSI guidelines. The susceptibility of isolates was determined for different antibiotics representing the important classes including co-amoxiclav, cefixime, penicillin G, oxacillin, ceftriaxone, erythromycin, levofloxacin, ciprofloxacin and meropenem.

**Results:**

Isolates were found to be mostly (82.3%) Gram positive (*Staphylococcus aureus* and *Streptococcus pyogenes*). *Pseudomonas aeruginosa* was the most abundant (17.9%) Gram-negative bacteria. *S. aureus* isolates were found to be 100% susceptible to levofloxacin, ciprofloxacin and meropenem. *S. aureus* exhibited less susceptibility to ceftriaxone (75%), and the least susceptibility to co-amoxiclav (37.5%), cefixime (31.3%), oxacillin (18.8) and erythromycin (25%). *S. pyogenes* isolates were found to be completely susceptible (100%) to co-amoxiclav, levofloxacin, ciprofloxacin and ceftriaxone. *S. pyogenes* had less susceptibility to vancomycin (92%), and the least susceptibility to cefixime (38.5%) and erythromycin (26.9%). *P. aeruginosa* isolates were found to be 100% susceptible to levofloxacin, ciprofloxacin and ceftriaxone, with less susceptibility to ceftriaxone (66%) and the least susceptibility to co-amoxiclav (22.2%), cefixime (33.3%), erythromycin (11.1%), vancomycin (11.1%) and oxacillin.

**Conclusions:**

Co-amoxiclav is extensively used in Sudan empirically. The results show that *S. aureus* and *P. aeruginosa* are increasingly becoming resistant to this drug. However, *S. pyogenes* is still susceptible to it. Conversely resistance to erythromycin and cephalosporins is shown in all the different isolated species, however these drugs are still widely used without even culture and ASTs. Luckily, non-susceptibility to ciprofloxacin and levofloxacin was not detected here. Obviously, laboratory identification and ASTs are much needed along with surveillance programmes in hospitals. Together with rational use of antibiotics these measures will help to slow down the progress of the deadly AMR pandemic.

